# A review of patients presenting to accident and emergency department with deliberate self-harm, KwaZulu-Natal, South Africa

**DOI:** 10.4102/phcfm.v9i1.1234

**Published:** 2017-05-29

**Authors:** Josephat O. Ani, Andrew J. Ross, Laura M. Campbell

**Affiliations:** 1 Department of Family Medicine, University of KwaZulu-Natal, South Africa

## Abstract

**Background:**

The World Health Organization has described deliberate self-harm (DSH) as a major global health challenge. Little is known about the profile of patients admitted following DSH at district and regional combo hospitals in KwaZulu-Natal, South Africa.

**Aim:**

The aim of this study was to assess the profiles of patients and reasons for admission following DSH.

**Setting:**

The study was conducted on data from a busy Accident and Emergency (A&E) department in a combination district and regional hospital situated in Empangeni in northern KwaZulu-Natal.

**Method:**

This was a retrospective descriptive study. Data were collected from charts of all patients admitted to the A&E department from April 2012 to March 2013 following DSH. Variables assessed included age, gender, race, occupation, religion, education level, coexisting medical and mental health conditions, and reasons for DSH. Data were entered into SPSS and analysed descriptively.

**Results:**

A total of 262 charts were identified and 215 (82%) were selected for inclusion. Most patients admitted following DSH were young, single African women with at least secondary-level education. Most (169/215;78%) admissions were for parasuicide, with relational issues contributing in more than 50% of cases and circumstance challenges contributing in just under 30%.

**Conclusion:**

Although an underestimation, DSH is not an uncommon reason for patients to present in the A&E at this district and regional combo hospital. Findings from this study are consistent with those of other studies on DSH and highlight the need for a validated screening tool for the identification of patients at risk of DSH. There is a need to explore community-based intervention, which could address reasons for DSH and prevent future admissions.

## Introduction

Globally, approximately one million people die from suicide each year,^1^ a number that exceeds deaths due to homicide and war combined.^1,2^ It has been estimated that for every actual suicide, an additional 20 people attempt to commit suicide.^3,4,5^ This is equivalent to an attempted suicide every 1 to 3 s and a successful suicide every 40 s.^4^ Between 9.5% and 11% of all non-natural deaths in South Africa are suicide related.^6,7^ In 2007, 3428 people committed suicide in South Africa,^8,9^ and the current suicide rate in the country is estimated at 13.4 per 100 000 members of the population.^10^ With such alarming numbers, deliberate self-harm (DSH) has been recognised as a global health challenge, and the World Health Organization (WHO) has issued a clarion call for action.^4^ Despite this call, there has been little urgency in identifying and preventing DSH.^4,11^

Deliberate self-harm includes suicide, attempted suicide and parasuicide.^12^ Suicide refers to DSH with planned action intended to cause death^13,14^; if that action is successful, then the patient has committed suicide. If that effort is thwarted, then the case is referred to as an attempted suicide. In contrast, parasuicide refers to a more impulsive action, the intention of which is to gain attention and seek assistance.^13,15^ The distinction is important, as many patients who present following DSH may actually be ambivalent about their wish to die, and the attempt may result from a strong wish to live and a need to communicate a plea for help.^13,16^

Both attempted suicide and parasuicide have been identified as important predicators for a future suicide.^15,17,18^ More than 2% of those who attempted suicide die from suicide within 1 year and more than 7% die within 10 years, whereas 40% of those who commit suicide have a past history of parasuicide.^11,13,19,20,21^

Literature reports that 10% of all those who commit suicide had been seen by a health care professional (HCP) in an Accident and Emergency (A&E) department within the preceding two months, but had not been identified as at suicide risk or assessed for suicide risk.^22^ This is due in part to the large number of patients presenting for emergency care at A&E departments and the fact that there are no clear screening tools that can be used regularly.^23,24^ These circumstances make it challenging for HCPs to identify patients at risk for suicide and attempted suicide accurately.

It is, therefore, important for HCPs to be aware of the common methods of DSH to ensure the appropriate management of patients presenting to an A&E department. Understanding the profile of patients who present to the A&E department following DSH may also help in recognising those at risk of suicide, as well as those using DSH as a ‘cry for help’. Although admissions for DSH are a common occurrence at many hospitals in South Africa, there is limited information about the profile of patients admitted to district and regional combo hospitals^1^ in KwaZulu-Natal (KZN) following DSH. There is correspondingly little data around common methods used and patient outcomes. This study sought to address this gap by focusing on patients admitted to an A&E department in KZN following an acute episode of DSH.

## Methods

This was a descriptive, retrospective cross-sectional study of the files of patients admitted to the hospital between 1 April 2012 and 31 March 2013 following DSH. The objectives were threefold: (1) to review the demographic profile of patients who presented to A&E after DSH, (2) to review reasons given for DSH and (3) to review patients’ methods selected for DSH. The intention was not to produce generalisable findings but to use the data to plan for screening and intervention. Data will also be used to educate staff in A&E departments and will guide further study in this important area.

The study site was a busy A&E department in a district and regional combination hospital situated in Empangeni in northern KZN. This hospital serves a large urban, peri-urban and rural population. It was purposively selected as a busy site in a very impoverished area of the country, where there are few formal employment opportunities, and poverty is a challenge faced by many. Many families rely on subsistence farming, social grants and income sent home by family members who work elsewhere.

Deliberate self-harm was taken to mean suicide, attempted suicide and parasuicide. All patients admitted to the hospital following DSH were recorded in the casualty register or resuscitation unit register before being transferred to an inpatient ward, psychiatric ward or resuscitation unit. No sampling methods were employed and the study population was all patients admitted to the A&E department with DSH during the study period. The names and hospital numbers of all patients admitted were retrieved from the casualty or resuscitation register, and the files were collected from the registry and analysed.

A data collection tool based on the study by Malangu and Ogunbanjo^25^ in Limpopo province was used to collect data from the retrieved records. The data collected included age, gender, race, occupation and religion, education status, background medical condition, methods, reasons, and an assessment of whether this was attempted suicide or parasuicide and help-seeking behaviour based on details provided in the notes. Data were also collected on treatment given and follow-up plans. Those patients who died in hospital following admission for DSH were recorded as having committed suicide. Data were entered into SPSS and analysed descriptively using frequencies. Chi-square tests were used to describe associations between variables, with a *p*-value <0.05 indicating significant association.

### Ethical consideration

Permission to conduct this study was given by the Biomedical Research Ethics Committee of the University of KwaZulu-Natal (BE218/13), the District Health Office, the Provincial Department of Health and the management of the district and regional combo hospital where the study was conducted, as well as the individual heads of relevant wards in the hospital. The study was anonymous as a number code was used to identify patient files.

## Results

A total of 262 charts were identified in the register as due to DSH during the study period. Two hundred and fifteen of these (82%) were included in the study as the remainder were found to have gaps in information around the study variables. Most DSH cases were recorded as parasuicide (169; 79 %), 33 (15%) were classified as attempted suicide and 13 (6%) were recorded as suicides (13; 6%). Although treatment given was included in the data collection sheet, this was not analysed because of insufficient information being available in the files.

The results are presented under the following headings: demographic profile, reasons given for DSH and methods used for DSH.

### Demographic profile

More women (144/215; 67%) than men (71/215; 33%) were admitted to A&E department with DSH. More women than men were also noted as parasuicide (122/169; 72%). An almost equal proportion of men and women attempted suicide (17 men and 16 women). More men than women committed suicide (8 and 5, respectively).

Ages ranged from 15 years to over 50 years but peaked in the 21–30 years age group, with DSH in the 15–30 years age group representing 77% (166/215) of the total number of patients admitted. Most parasuicide occurred in the 15–30 years age group, which represented 84% (139/169) of the total number of patients admitted following parasuicide.

Attempted suicides were fairly consistent over all age groups. Suicide was most common in the age group 21–30 years (53%). There was a significant association between age and type of DSH (*p* < 0.01), with parasuicide and attempted suicide being more prevalent in younger age groups. A significant association was found between gender and type of DSH (*p* < 0.01), with more women than men recorded as parasuicide and attempted suicide cases.

Most patients admitted following DSH were described as African (186; 87%), and the remainder as White (18; 8%) or Indian (11; 5%). Most were single (159; 74%), with the remainder being married (34; 16%), divorced (21; 10%) or widowed (1; 0.5%). A significant association was found between marital status and the type of DSH (*p* < 0.01), with single people being more likely than other groups to be admitted with parasuicide, attempted suicide and suicide. Further demographic details are provided in Table 1.

**TABLE 1:** Demographic profile of patients admitted to the hospital following an episode of deliberate self-harm.

Patient characteristics	Total, *N* = 215 (frequency %)	Parasuicide, *n* = 169 (78.60 %)	Attempted suicide, *n* = 33 (15.35 %)	Completed suicide, *n* = 13 (6.05 %)	Pearson’s chi-square (*X*^2^) test
Gender	-	-	-	-	10.4221
Female	144 (66.98)	122 (72.19)	17 (51.52)	5 (38.46)	-
Male	71 (33.02)	47 (27.81)	16 (48.48)	8 (61.54)	-
Race	-	-	-	-	2.654.0
Black	186 (86.51)	149 (88.17)	27 (81.82)	10 (76.92)	-
White	18 (8.37)	13 (7.69)	3 (9.09)	2 (15.38)	-
Indian	11 (5.12)	7 (4.14)	3 (9.09)	1 (7.69)	-
Age group (years) (mean age = 26.38 ± 9.1 SD)	-	-	-	-	24.1363
15–20	59 (27.44)	57 (27.51)	2 (0.93)	0.00	-
21–30	107 (49.77)	82 (38.14)	18 (8.37)	7 (3.26)	-
31–40	32 (14.88)	21 (9.77)	8 (3.72)	3 (1.40)	-
41–50	10 (4.65)	6 (2.79)	2 (0.93)	2 (0.93)	-
>51	7 (3.26)	3 (1.4)	3 (1.40)	1 (0.47)	-
Education level	-	-	-	-	14.8969
None	41 (19.07)	27 (15.98)	11 (33.33)	3 (23.08)	-
Primary	31 (14.42)	22 (13.02)	5 (15.15)	4 (30.77)	-
Secondary	118 (54.88)	103 (60.95)	12 (36.36)	3 (23.08)	-
Tertiary	25 (11.63)	17 (10.06)	5 (15.15)	3 (23.08)	-
Marital status	-	-	-	-	22.138.0
Divorced	21 (9.77)	12 (7.10)	6 (18.18)	3 (23.08)	-
Married	34 (15.81)	19 (11.24)	11 (33.33)	4 (30.77)	-
Single	159 (73.95)	137 (81.07)	16 (48.48)	6 (46.15)	-
Widow	1 (0.47)	1 (0.59)	0.00	0.00	-
Occupation history	-	-	-	-	32.5301
Pensioner	3 (1.40)	2 (1.18)	1 (3.03)	0.00	-
Professional	11 (5.12)	7 (4.14)	3 (9.09)	1 (7.69)	-
Skilled work	26 (12.09)	12 (7.10)	10 (30.30)	4 (30.77)	-
Student	56 (26.05)	55 (32.54)	1 (3.03)	0.00	-
Unemployed	84 (39.07)	67 (39.64)	11 (33.33)	6 (46.15)	-
Unskilled work	35 (16.28)	26 (15.38)	7 (21.21)	2 (15.38)	-
Religion	-	-	-	-	8.8319
African traditional	26 (12.09)	20 (11.83)	3 (9.09)	3 (23.08)	-
Christian	87 (40.47)	74 (43.79)	10 (30.30)	3 (23.08)	-
Hindu	9 (4.19)	5 (2.96)	3 (9.09)	1 (7.69)	-
Islam	1 (0.47)	1 (0.59)	0.00	0.00	-
No religion	52 (24.19	40 (23.67)	8 (24.24)	4 (30.77)	-
Zionist	40 (18.60)	29 (17.16)	9 (27.27)	2 (15.38)	-

The level of education varied between none to tertiary education. There was a significant association between level of education and type of DSH (*p* = 0.02). Those with secondary school level had more frequent DSH (parasuicide and attempted suicide) than those with other levels of education.

Over a third of those admitted were unemployed (84; 39%); the remainder were described as follows: students (56; 26%), skilled (26; 12%), unskilled (35; 16%), professionals (11; 5%) and pensioners (3; 1.5%). There was a significant association between type of DSH and occupation (*p* < 0.01), with those who were unemployed being more likely to be admitted because of parasuicide, attempted suicide and suicide.

### Reasons provided for deliberate self-harm

Reasons provided for DSH were categorised into four groups: (1) relationship problems, (2) circumstance challenges (e.g. unemployment, rape, retrenchment and pregnancy), (3) medical problems (bipolar, HIV infection, multidrug-resistant tuberculosis, infertility and impotence) and (4) persons not really sure of the reason (Table 2).

**TABLE 2:** Reasons given^a^ and type of suicide.

Reasons	Type of suicide			Total (*n* = 215)
	Parasuicide (*n* = 169)	Attempted suicide (*n* = 33)	Completed suicide (*n* = 13)	
Relationship issues	97	15	1	113
Circumstance challenges	43	11	10	64
Medical problems	8	2	1	11
No reason given	21	5	1	27

a Some patients gave more than one reason.

The reason most often given for DSH was relationship challenges (52.5%; 113/215). In parasuicide, relationship challenges accounted for 57% (97/169) of admissions. In attempted suicide, relationship problems were responsible for 45% (15/33) of admissions, whereas in suicide, relationship challenges were reported to account for only 8% of admissions. Details of reasons given are presented in Table 2. Most people who carried out DSH did not report a mental health disorder, with only 7 reporting a mood disorder, 2 being diagnosed with schizophrenia, 2 having bipolar disorder and 40 who were HIV positive (Table 3).

**TABLE 3:** Medical problems reported at time of admission for deliberate self-harm.

Medical condition	Type of deliberate self-harm			Total
	Parasuicide (*n* = 169)	Attempted suicide (*n* = 33)	Completed suicide (*n* = 13)	
Hypertension	4	3	2	9
Diabetes	1	2	1	4
Epilepsy	2	0	0	2
HIV positive	27	8	5	40
Major depressive disorder	5	2	0	7
Schizophrenia	1	1	0	2
Bipolar disorder	2	0	0	2
**Total**	**42**	**16**	**8**	**66**

### Methods used

Methods used for DSH included attempted hanging, use of a firearm, medication, organophosphates, herbal poisoning, household bleach and a stab to abdomen. The relative proportions of each method used in DSH are shown in Table 4.

**TABLE 4:** Methods used and types of deliberate self-harm.

Methods used for deliberate self-harm	Type of deliberate self-harm			Total
	Parasuicide (*n* = 169)	Attempted suicide^b^ (*n* = 34)	Completed suicide (*n* = 12)	
Hanging	3	5	5	13
Firearm	0	0	1	1
Medication	132	20	1	154
Organophosphates	22	6	4	32
Other^a^	12	2	1	15

a Other includes herbal poisoning, household bleach, stab to abdomen.

b Some data missing.

There was a wide range of medications used in DSH, with the three most common being paracetamol (113; 86% of all medications involved paracetamol), antiretrovirals (54 patients) and organophosphates (32 patients). Fifty-six patients admitted having taken multiple medications.

Other substances that were reported to be present at the time of DSH included alcohol, cigarettes, dagga, wunga, cocaine and heroin. The relative proportions of these are represented in Table 5.

**TABLE 5:** Other substances used at the time of deliberate self-harm.

Substance	Alcohol	Cigarettes	Dagga	Wunga	Cocaine	Heroin
Parasuicide, *n* = 169	62	15	5	4	7	2
Attempted suicide, *n* = 34	20	4	2	2	1	2
Suicide, *n* = 12	6	4	2	2	2	1

Eleven patients were referred to a psychiatrist, 63 to a psychologist and 84 to a social worker prior to discharge. Follow-up records from these departments were not available for review.

## Discussion

The aim of this study was to add to information around people who present to A&E department with DSH at a district and regional combo hospital in KZN. A greater understanding of the antecedents to DSH is important in order to identify high-risk groups and to develop effective prevention and intervention strategies. Local studies on DSH are useful as literature indicates that national surveillance data around suicide may greatly underestimate the problem.^26^

It was encouraging that 82% of charts contained sufficient information for inclusion in the study. This speaks positively of record-keeping quality in a busy South African A&E. The importance of good record-keeping is stressed as vital for the well-being of the DSH patient and also to protect the clinician against potential future litigation.^27^ More women than men presented with DSH, and this finding is in keeping with those of other studies on DSH in South Africa.^28^

This study only reviewed patients with DSH who presented to A&E department, which may be an underestimation of the problem of DSH, as there may be many more people who commit DSH in the community without ever reaching an A&E department. As an example, a community-based study in urban KZN revealed that 38% of the participants had probable depression and high numbers of women reported suicide ideation in the previous seven days (145/380; 38.3%).^29^ In that study, researchers found a significant association between probable depression and suicide ideation, with risk factors including being single and without support, being HIV positive, having an unplanned pregnancy and a past history of depression.^29^

A community-based study may also be required to look at the gender bias in DSH among women in A&E setting: men may not tend to report to A&E after an episode of DSH or may die prior to reaching a hospital. The attitudes of isiZulu-speaking people (the majority of the population) regarding DSH are largely unknown, and there may be severe underreporting of DSH.

The largest age group for DSH in this study was relatively young, with a mean age of 26.38 years. A WHO report released in 2012 identified young people as increasingly vulnerable to DSH, with suicide reported as the second leading cause of death in those aged between 15 and 19 years.^11^ Although the mean age of admission in this study was slightly older than that of the WHO report, the findings from this study support other Southern African studies, which have documented that the majority of DSH occurs in the 20–30-years age group.^28,30^ However, these data may require further investigation, as other South African literature demonstrate that the frequency of DSH increases with increasing age.^31^ There was a significant association between gender and type of DSH (*p* < 0.01), with more women than men recorded as parasuicide and attempted suicide cases, which is consistent with other South African studies.^25^

Marriage was found to be protective, with less suicide and attempted suicide among those who were married, which is similar to the findings of other studies.^28,31^ However, with the small number of patients admitted following suicide and attempted suicide, these figures must be treated with caution. In addition, it is also difficult to comment on the frequency of DSH and race, as there are proportionately more Africans attending this hospital than Whites or Indians because of its location and the population accessing health care here. This would be a useful area for future study.

Secondary-level education was associated with increased frequency of DSH, particularly among those admitted following parasuicide and attempted suicide. This finding also requires further investigation, as other studies report that DSH – including that leading to suicide – is higher among professionals such as doctors, dentists and police officers.^26^

This study supports a common finding that being employed generally protects against DSH and suicide.^28,31^ Religious belief also seems to be important when assessing DSH. South African literature reports that historically Roman Catholics had lower suicide rates compared with other religious groups such as Protestants or Jews.^18^ However, having any strong religious belief is a protective factor against DSH.^18^

A major finding was that most patients presenting with DSH did not have any diagnosis of mental health disorder; only 11 patients had a recorded psychiatric diagnosis, and this requires further exploration. This may be related to the relatively young age at presentation, as younger people may not have had psychiatric evaluation and diagnosis at an early stage of mental health illness, or the finding may be related to the lack of mental health services available. Other studies illustrate that a psychiatric diagnosis is strongly associated with DSH,^12^ with studies in Africa reporting that 90% of those who commit suicide have a diagnosis of mental health disorder.^32^ Recent research has suggested involving traditional healers in screening for and educating around mental health disorders, and that there may be a place for involving African healers in any DSH intervention.^15,33^ Community-based screening and intervention for DSH and mental health problems may be worth consideration as part of this hospital’s outreach programme.

The study did not consider other factors that may have been relevant in reviewing DSH, for example place of residence. A South African study indicated that rural people were much more likely to commit suicide than urban dwellers.^34^ Although there have been great efforts to heal the atrocities of apartheid, many challenges remain, especially in the area of Zululand in northern KZN, where this study was conducted. Poverty in the area is still systemic, employment opportunities are scarce, education can be rudimentary and fragmented, and health care, including mental health care, may be inaccessible and unaffordable. Violence is endemic. Television news, newspaper articles and social media compound pervasively negative discourses around the future of South Africa and of the world. People who see no productive future and who are mentally ill or physically harmed may become deeply disillusioned and may choose DSH.^12^

Factors associated with admission included alcohol consumption with over 40% of those admitted having consumed alcohol at the time of DSH, a finding that is supported by other studies.^34,35^ The most common reason recorded for DSH was around relationship challenges, and this is congruent with other South African studies.^28,32^

Paracetamol was the most common substance used as medication for DSH, and this finding was consistent with other studies.^36^ This requires further investigation as people may be unaware of the potential dangers of paracetamol. Increasing awareness around the dangers of alcohol and paracetamol may be an immediate intervention in a busy A&E department.

Although organophosphates were used by 15% (32/215) of those who carried out DSH, further study is required as findings from other reviews suggest that pesticide poisoning is the most prominent method of DSH in South Africa.^36^ Moreover, the proportion of DSH involving pesticides is likely to be underestimated, as data are largely absent in rural areas where pesticides are easily accessible and likely to be a commonly used DSH method.^36^

It was disappointing to note that in this study, only 73% (158/215) of patients admitted following DSH were referred for psychosocial support, particularly as an admission represents an opportunity to address factors that may be contributing to suicide ideation and intent. Reasons for this need to be explored further.

## Limitations to the study

An assumption was made that data entered onto the register and into patient’s charts were correct, and further research may look at the accuracy of such records. The study did not intend to be generalisable, and in any future study, data validity could be triangulated by replicating the study in differing sites and by using differing data collection methods, such as sentinel data collection sites or point prevalence and incidence studies. Qualitative review would add richness to the study by exploring the underlying reasons why people choose DSH and what they consider could be of help to them.

## Conclusions

This timeous study illustrated that DSH is not an uncommon cause of admission to A&E department, and patients are generally young, female, unmarried and African with secondary-level education. There are many unanswered questions, as admissions may represent the tip of the iceberg with many more people at risk in the community.

The attitudes of African isiZulu-speaking people to DSH are largely unknown, and this requires qualitative review to assess whether there is reluctance to report DSH intention. Community-based intervention would perhaps prevent admissions with DSH to A&E departments.

Although easily available, because of the low sensitivity and low predictive value of the ‘sad person score’,^23^ the National Institute for Health and Care Excellence guideline update in April 2013 recommended that the sad person score not be used as a screening tool to help in identification of those at risk of DSH presenting to A&E department.^24^ In the light of this, it would be worth investigating the effect of introducing another tool for compulsory screening of all patients seen in the A&E department such as the suicide risk guide^22^ to help in the identification of patients who might be at risk of DSH.

## Footnotes

1 A district or regional combo hospital is a hospital that provides both first-level generalist care and care provided by teams led by resident specialists.
